# Ruptured benign thyroid nodule post-radiofrequency ablation

**DOI:** 10.1210/jcemcr/luag321

**Published:** 2026-10-29

**Authors:** Corina Dang, Agustin A Andrade

**Affiliations:** Florida International University Herbert Wertheim College of Medicine, Miami, FL 33199, USA; Florida International University Herbert Wertheim College of Medicine, Miami, FL 33199, USA

**Keywords:** thyroid rupture, radiofrequency ablation, thyroid nodule

## Image legend

A 69-year-old male presented to the thyroid clinic with a 7.7-cm (92 mL) nodule (Panels A-C). Two fine-needle aspirations revealed benign cytology, but due to symptoms of pressure and aesthetic discomfort, the patient opted for radiofrequency ablation (RFA) and declined surgery. The RFA procedure, using Starmed equipment with a 2-cm tip, was uncomplicated. Two months later, he presented with nodular cutaneous fistulization of new cystic content; a diagnosis of thyroid nodule rupture was confirmed via ultrasound (Panels D and E). Fine-needle aspiration was attempted with minimal yield and a negative culture. He was then treated with prophylactic antibiotics (doxycycline) and corticosteroids for 30 days with complete symptom and fistula resolution. Seventeen months later, ultrasound showed a 5.2-cm (31.8 mL) nodule, corresponding to a 65% reduction. Twenty-two months after treatment, the nodule measured 4.0 cm (19.9 mL), corresponding to a 78% reduction (Panel F). The patient was very satisfied with the outcome (Panel G). While all patients in our clinic (RFA-thyroid nodule rupture incidence 0.4% [0.2%-2.5% in the United States]) underwent conservative management with >50% nodule reduction, sometimes surgical intervention may be necessary [1, 2]. It is imperative that physicians are familiar with this post-RFA complication to avoid unnecessary procedural intervention and provide reassurance to the patients.

**Figure:**
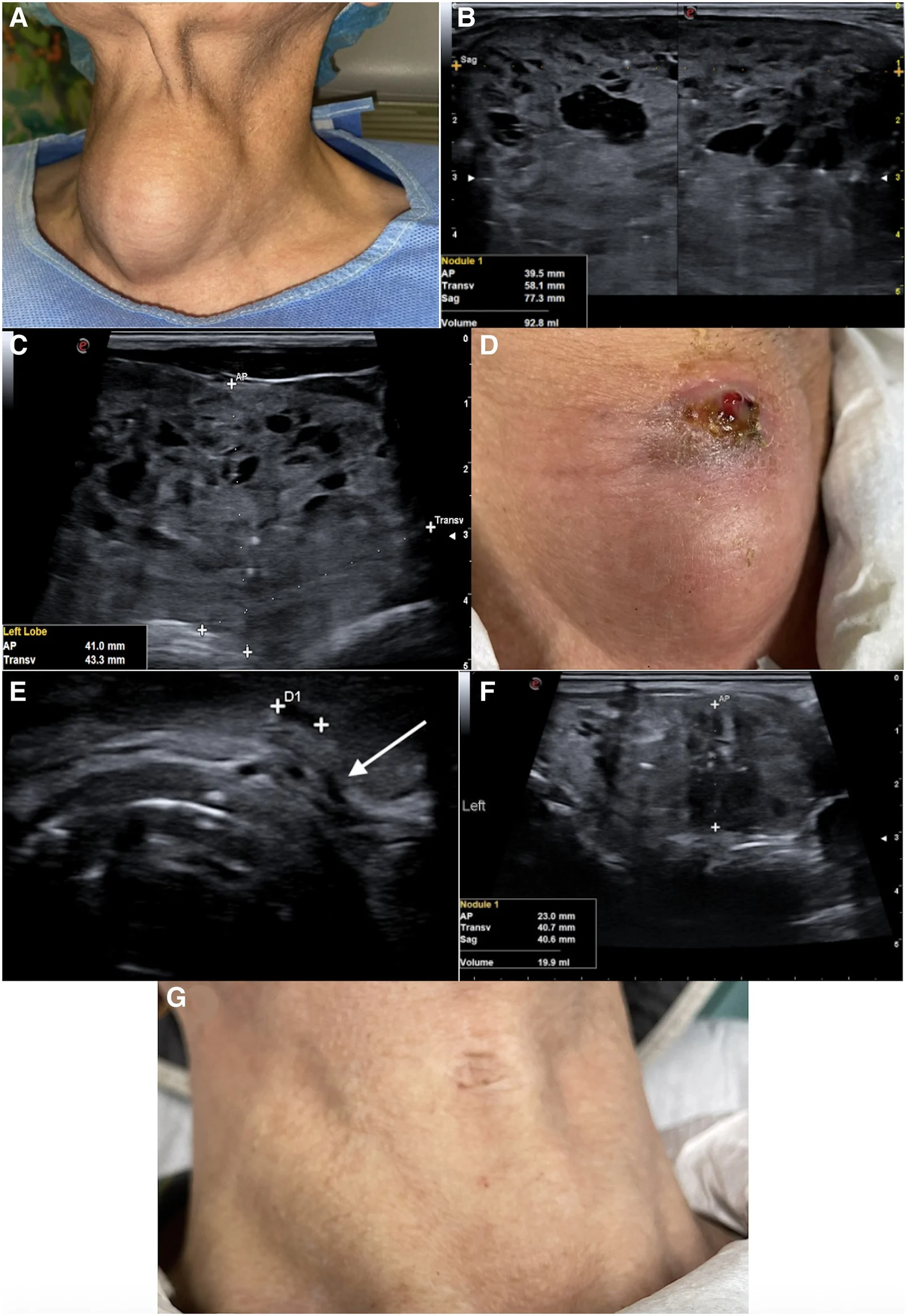


## Data Availability

Data sharing is not applicable to this article as no datasets were generated or analyzed during the current study.
